# Monthly Summary

**Published:** 1891-02

**Authors:** 


### Monthly Summary.

PRACTICAL SUGGESTIONS.—*By Dr. E. E. Shattuck, Kansas City.*—One of the most important things to consider, is our dental office. It is there we pass the best part of our life. Our failure or success in a business way depends more on our surroundings than most of us are aware. Intelligent and cultured people look to the environments on entering an office when calling to make the first appointment, and your ability as a dentist will be very correctly gauged by their first impression. It need not necessarily be furnished expensively. The one thing needful is to have it clean and neat in every detail. It should have a pleasant, inviting appearance, and tastefully arranged. This has an elevating influence on the character and disposition of the dentist. He is inclined to be more cheerful and patient with nervous and exacting patrons. Do not be afraid of spending too much money in furnishing your office. It will be a good investment. I never saw a clean, well-arranged dental office but the dentist himself was neat and particular in his appearance, and also a first-class workman. I can always tell what kind of a dentist he is by his office, or the kind of an office by the dentist. We ought not to consider this subject in regard to a well-kept office of little or no importance. It will always bring a better class of patients who are willing to pay the price we ask, and this is a stimulant for us to excel in each succeeding operation. It is a duty we owe to the profession to do all we can to improve ourselves in dentistry.

Before beginning an examination, or operating on the teeth, always wash your hands, and be sure your finger nails are clean. Unclean finger nails are disgusting under all circumstances. Always use a clean napkin or towel for each patient. Always use a new piece of rubber dam for each patient at every sitting. Do not save the same piece to be used at their next engagement. They will have their doubts about its being the same one they had before. It makes a bad impression and adds but little to our profits at the end of the year. Always use as thin rubber dam as possible. Use fine floss silk, or linen thread well waxed. Soap the dam where you have punched the holes for the teeth; you will be surprised how easily it is adjusted where the teeth are crowded. Use cocaine on the gums around the anterior teeth. The patient will lose all dread while applying the dam, and in furnishing approximal cavities the operation will be painless. Always dry out the cavity before commencing to excavate, to enable you to see in what direction the decay has extended. All operations should be made as painless as possible.—*Archives of Dentistry*.

---

THE NATURE OF KOCH'S LYMPH.—Prof. Koch has made a partial statement as to the composition of his lymph. From the beginning of the treatment of tuberculous affections with this fluid, its composition has been an open secret. There is nothing in the recent statements of Prof. Koch that will lead any one to be much wiser on the subject than they previously were. The simple statement that the lymph consists of a glycerine extract derived from the pure cultivation of tubercle bacilli does not enable one to have very clear ideas as to the nature of the agent. Prof. Koch says: "Into the simple extract there naturally passes from the tubercular bacilli, besides the effective substance, all the other matter soluble in 50 per cent. of glycerine, consequently it contains a certain quantity of mineral salts, coloring substances, and other unknown extractive matters. Some of these substances can be removed from it tolerably easily. The effective substance is insoluble in absolute alcohol. It can be precipitated by it, though not, indeed, in a pure condition, but still combined with the other extractive matter. The coloring matter may also be removed, rendering it possible to obtain from the extract a colorless, dry substance containing the effective principle in a much more concentrated form than the original glycerine solution. For application in practice this purification of the glycerine extract offers no advantage, because the substances so eliminated are unessential for the human organism."

Prof. Koch himself appears to have no definite knowledge as to the nature of his lymph..—*Montreal Med. Journal.*

---

COFFEE AS A DISINFECTANT.—The *Deutsche Medizinal-Zeitung* for September 22, 1890, quotes the following from the *Journal de Medecine d' Algerie.* On an extremely hot day in Algiers, Dr. Barbier and some officers were directed to a village inn, to make an examination of the body of a murdered man. They were led to a small apartment in which the body lay, but were met by so intolerable an atmosphere that they at once retreated. Upon request a plate of ground coffee was brought and sprinkled upon the cadaver, the floor and the walls. The horrible stench disappeared and the examination was proceeded with without interruption. Gratified with the result, Barbier soon found occasion to make a similar use of coffee, with equal success, in the case of a body which had lain in the water for a week. He now began to use coffee in this way in the treatment of foul ulcers, and with good results. He considers coffee an antagonist of the narcotic effects of tobacco, for which there is no better antidote. He also recommends the use of coffee for the disagreeable odor sometimes acquired by ice-chests for the preservation of animal foods. To preserve game, this should be first cleaned and then strewn with coffee. It was also observed that cholera bacilli and other microbes lose the faculty of multiplication in infusions of coffee.—*Med. and Surg. Reporter.*

CHLOROFORM AS AN ANTISEPTIC.—The *Lancet*, December 20, 1890, says, Salkowski has demonstrated as the results of his experiments that chloroform prevented fermentation and decomposition in milk and other fluids. M. Kichner (*Zeitschr. fur Hygiene*, viii, 1890, Heft 3) has also made some investigations in regard to this property of chloroform. He first showed that, when mixed with blood serum, no decomposition of the latter occurred, and then further showed the same action on milk, and also its purifying properties on waters known to be rich in bacteria, and again, on pure cultures of pathogenic and non-pathogenic organisms. The method employed was as follows: Chloroform was added in excess to the fluid to be experimented on; the vessel in which it was contained was then hermetically sealed and thoroughly shaken. The various specimens were kept at different temperatures—namely, that of body or air, or in an ice chamber. The contents were tested after variable periods of time, lasting from hours to years, by means of plate cultivations. The results obtained were that chloroform has a powerful destructive action upon a great number of bacteria, but does not kill their spores. The bacilli of anthrax, cholera and enteric fever, as well as the staphylococcus aureus, were quickly destroyed by chloroform. The spores of the anthrax, and tetanus bacilli were not affected by the drug. In spite of its presence the spores developed into bacilli, which were then rapidly killed. Kirchner considers from these results that milk to which chloroform has been added can be preserved for a long period, although we cannot be sure that it is free from spores which will eventually develop. Kirchner recommends its use for the preservation of fluids containing albumin, and especially for sterilizing blood serum, the composition and coagulating power of which are not appreciably altered by its presence, and its use as a cultivating medium is not influenced. The mixed fluids are best preserved at the ordinary temperature of a room and not in an ice chamber. Kirchner further recommends chloroform water for the disinfection of wounds, and states that it is especially useful in gynecological and obstetric practice, and also for the disinfection of bed-pans, linen, etc., in epidemics of cholera and typhoid fever. He supports Salkowski's views as regards its favorable action on some intestinal affections, especially the diarrhœa of children. He also advises that it should be used in military campaigns for the purification of drinking water.—*Med. and Surg. Reporter.*

---

PHYSIOLOGICAL ACTION OF THIALDINE.—Thaldine, obtained for the first time by Woehler and Liebig, in the form of large crystals, of aromatic odor, which volatilize at the ordinary temperature without decomposing, is but slightly soluble in water, dissolves rapidly in alcohol and acids. According to *Les Nouveaux Remedes* for November 8, 1890, from experiments made with this substance the following conclusions were reached:

1. Thialdine is very poisonous in frogs in the dose of 1-3 grain for every one hundred and fifty grains body-weight, though much less poisonous in rabbits.

2. Its administration is followed in the frog by profound sleep, with loss of general and reflex sensibility, depressed sensibility of the motor nerves, while the muscular irritability remains intact. In the rabbit the anæsthesia is less intense, and interrupted by excitation.

3. Thialdine invariably causes slowing of the pulsations of the heart, which, nevertheless remain perfectly regular. It is only when given in very large doses, and then only immediately before death, that the pulsations of the heart become irregular.

4. When given in small doses, the slowing of the pulse is only transient. Injected in small doses in the excised heart, thialdine diminishes the number of pulsations, at first prolonging the diastole, after rendering the contractions of the heart irregular and more rapid, and finally arresting the heart in diastole.

3. In large doses it slows at the outset cardiac contractions, and then leads to the arrest of the heart in diastole.

It would thus seem that carbo-thialdine and thialdine differ absolutely from each other as regards their physiological action. The first is an energetic tetanizer, and never produces irregularity of the heart, which it arrests in diastole. On the other hand, thialdine produces irregularity of the heart which, in the majority of cases, it arrests in systole; finally, while carbo-thialdine produces no effect on rabbits, thialdine is poisonous in them, though to a less degree than in frogs.—*Therapeutic Gazette*, January, 1891.

---

A READY INHALER.—In the *British Medical Journal*, November 22, 1890, Mr. Arthur H. Rideal gives the following description of a ready method of arranging what he says is a most effective inhaler. Take an ordinary funnel, and in it loosely pack some cotton-wool. On this sprinkle the medication (ol. eucalypti., etc), and then tie a piece of gauze or muslin over the funnel to keep the wool in position. Invert the funnel over some hot water contained in a breakfast cup, or other vessel, according to the size of the funnel, and an inhaler is obtained which is equal, as regards its utility, to the most elaborate.

---

CURES FOR NEURALGIA OF THE HEAD.—An English physician has recently asserted that head-aches and neuralgia could be stopped by blowing a solution of salt up the nose. A Swiss physician, Dr. Naegely asserts that he can cure neuralgia of the trigeminus by pressing up the hyoid bone.—*Medical Record*.

CAMPHO-PHENIQUE.—Dr. J. W. Downey, writing on this antiseptic in *Archives of Dentistry*, says: Campho-phenique is a germicide and antiseptic. Campho-phenique is absolutely free from toxic or caustic properties. Applied to the unbroken skin it produces no sensation whatever. On cut surfaces there is a slight burning sensation when first applied, followed by anæsthesia.

Now, which antiseptic is the most agreeable. The brassy metallic taste of the bichloride is intolerable, the taste and smell of carbolic acid and creosote are disagreeable to most people, and the odor and meagre antiseptic properties of iodoform should banish it from the operating-room. Campho-phenique has a pleasant odor and agreeable taste; this should establish its claim as the most agreeable germicide summing up, he says.

1st. That when used pure and undiluted, campho-phenique is one of the most efficient and reliable germicides and antiseptics.

2. Being non-poisonous and non-irritant, it is perfectly safe.

3. It is the most agreeable to the patient of any drug of its class.

As to special uses he advises it as a pulp canal dressing in the various pathological conditions, from recent devitalization to alveolar abscess. Here it will take the place of corrosive sublimate, carbolic acid, creosote, oil of cloves, iodoform, or any germicide heretofore used, except peroxide of hydrogen. If thoroughly rubbed on the gum, or injected with a hypodermic syringe, it acts efficiently as a local anæsthetic, not equal, however, to cocaine; but there are no constitutional effects following its use, and there is no danger of the tissues sloughing. It is quite efficient as an obtunder of sensitive dentine.

The very disagreeable ache which sometimes follows the extraction of abscessed teeth is almost instantly relieved by placing a pledget of absorbent cotton, saturated with campho-phenique, deep in the painful socket.

For chapped hands the following formula is advised :—

R—Campho-phenique - - -
Oil of cade - - - - aa ʒj
Rose cosmoline - - - - ℥j
m. Sig.—Apply frequently.

Campho phenique should never be mixed with water or glycerine. It will mix in all proportions with alcohol, ether, chloroform, and all fatty substances. In dentistry it will seldom be necessary to dilute it at all.—*London Dental Record.*

---

ADMINISTRATION OF MORPHINE BY THE NOSTRILS.—As a rule, where persons dislike the taste of morphia, or where it is desired to have a rapid physiological action, the hypodermic method of administration is resorted to. This method is open to the objection that it requires a small surgical operation and that this sometimes is followed by the formation of an abscess.

I desire now to describe a new method of administering morphia, namely, through the mucous membrane of the nose —a method which has been thoroughly tested by me in over one hundred cases, in which the immediate physiological action of morphia was necessary. I have found it to be reliable and more prompt in its action than the administration of the drug by the mouth or hypodermically. It requires much smaller doses than is requisite in d sing by the mouth, and no larger dose than is usual for hypodermic administration.

The manner of administering morphia through the olfactory canal is simply by causing it to be snuffed up the nasal chambers, in the same manner and in the same way as snuff tobacco is used. The dose is divided into two equal parts, and each part is placed upon the end of the thumb and snuffed up into the nostril. The membrane being very soft and delicate it appears to be absorbed instantly. I have tried it on myself on several occasions and the absorption seemed almost instantaneous. Fifteen seconds after the introduction of the morphia, I have blown my nose; and nothing could be found in the secretion. However, in poor qualities of morphia, the drug will not be absorbed as readily.

In cases where the nasal chambers are covered by crusts or dry secretion the cavities should be cleaned before administering morphia, care being taken not to snuff it up too strongly. If the drug is of a poor quality it will produce sneezing; but the narcotic effect will be the same.

When morphia is administered in the manner I have just described, it is tasteless, its action is rapid and the narcotic effect lasts for a longer period than when this drug is taken either by mouth or hypodermically.—CARL H. VON KLEIN, A. M., M. D., Dayton, Ohio; in *Med. and Surg. Reporter.*

---

CODEIN.—According to Lowenmeyer (*Deutsche medicinische Wechenschrift*, No. 20, 1890), codein is very little employed in Germany, notwithstanding the encomiums which it receives from physicians elsewhere and especially from the French. He therefore recently instituted an extensive series of observations in Jacobson's service at the Jewish Hospital in Berlin. Some five thousand doses of the drug were given to about four hundred patients, some of whom took it for weeks, and others for months; yet in no case did he observe any untoward results. He therefore recommends its use in place of morphine, rating it as a narcotic of somewhat lesser intensity than the latter, but on the whole superior, because of its freedom from danger. He especially endorses Dr. Lauder Brunton's assertion of the peculiar applicability of codein to the relief of painful affections of the abdomen. Gastralgia, colic, and the various visceral neuralgias seemed peculiarly amenable to its influence.—*Medical News.*

---

A NEW MIXTURE FOR USE IN LOCAL ANÆSTHESIA.—Dr. A. Dobisch, of Zwittau, has used, for the purpose of producing local anæsthesia, a spray, with Dr. Richardson's ether spray apparatus, composed of ten parts of chloroform, fifteen parts of sulphuric ether, and one part of menthol. After one minute's application of this spray complete anæsthesia of the skin and neighboring tissues were obtained, which lasted for from two to six minutes, and sufficed for the performance of such minor operations as opening abscesses of the cervical glands, incising a deeply seated whitlow, and the excision of an epithelioma of the nose. In all the cases in which he employed the spray above mentioned the wounds healed quite satisfactorily.—*The Lancet.*

---

ISOCOCAINE, A NEW ANÆSTHETIC.—The *Pharmaceutical Record*, October 20, 1890, says if ecgonin is treated with caustic, soda or potassa, it changes into the isomeric isoecgonin. If the chloride of this base be suspended in alcohol, in which it is insoluble, and hydrochloric acid gas be passed into the mixture, it becomes clear. When evaporated to dryness, the residue taken up with water, the solution made alkaline and shaken with chloroform, beautiful prismatic plates of isoecgonin ethylether separate. Isococaine is obtained from this by heating with two parts of benzoylchloride for a short time in oil, both up to 150° or 160°. The melting point of isococaine is at 44° C., that of cocaine at 98°. With hydrochloric, nitric and hydrosulphuric acids, isococaine forms sparingly soluble salts. The process for making it has been patented by the firm Bohringer, in Mannheim. Its advantage over cocaine is a quicker production of anæsthesia. The oculist cannot use it, as it is even more of a local irritant than cocaine.—*Medical and Surgical Reporter.*

